# OD30 Clinician-Driven Health Technology Assessment: National Cancer Medicines Review For Off-Label Uses And On-Label Off-Patent Uses In NHSScotland

**DOI:** 10.1017/S0266462324001612

**Published:** 2025-01-07

**Authors:** Richard O’Connell, Louis Doherty, Pamela Andrews, Louise Craig, James Drinkell, Heather Dalrymple, Sally Clive

## Abstract

**Introduction:**

Publicly funded cancer services face significant financial and capacity challenges. It is estimated that 40 percent of medicines used to treat cancer are outside their marketing authorization or “off-label.” These uses are usually outside the remit of health technology assessment (HTA) groups. Accessing emerging off-label uses is mostly through individual patient requests, which are resource intensive, delay patient treatment, and produce inequity.

**Methods:**

A program providing national HTA review of off-label and off-patent cancer medicine uses has been established by Healthcare Improvement Scotland. Processes include horizon scanning, support for proposing clinicians, and engaging patient groups. Relevant published and unpublished clinical and cost-effectiveness information—identified through systematic literature searches, engagement with pharmaceutical companies, academic and health service data groups—supports independent appraisal and decision-making. Where cost-effectiveness information is unavailable, a value-judgment framework, including magnitude of clinical benefit, uncaptured benefits, and budget and service impact, is utilized to standardize review. The decision-making Council includes public partners, and advice is shared across NHSScotland.

**Results:**

From July 2022 to October 2023, the program has published advice on nine proposals—eight off-label uses and one on-label off-patent use. Health economic models from a pharmaceutical company and an academic group supported decision-making on two proposals, value-judgment frameworks for two proposals, and real-world evidence for one proposal. Eight proposals were supported, and one was not supported. Each supported proposal slowed cancer, prolonged life, or reduced toxicity compared to standard treatment options. Four were cost-saving and three had a low medicines budget impact. Three were service-saving and three had no significant impact on services.

**Conclusions:**

Novel HTA programs can address gaps in medicines governance to improve patient outcomes and support sustainability. Clinical connections, patient group engagement, health economic collaborations and linkage to national cancer data teams and academics have facilitated bespoke approaches to evidence-gathering despite limited resources. Our agile and adaptive approach has enabled robust review and decision-making on varied and impactful proposals.

